# Evaluating Sex and Age Biases in Multimodal Large Language Models for Skin Disease Identification from Dermatoscopic Images

**DOI:** 10.34133/hds.0256

**Published:** 2025-04-01

**Authors:** Zhiyu Wan, Yuhang Guo, Shunxing Bao, Qian Wang, Bradley A. Malin

**Affiliations:** ^1^ Department of Biomedical Informatics, Vanderbilt University Medical Center, Nashville, TN, USA.; ^2^ School of Biomedical Engineering, ShanghaiTech University, Shanghai, China.; ^3^ Department of Electrical and Computer Engineering, Vanderbilt University, Nashville, TN, USA.; ^4^ Department of Computer Science, Vanderbilt University, Nashville, TN, USA.; ^5^ Department of Biostatistics, Vanderbilt University Medical Center, Nashville, TN, USA.

## Abstract

**Background:** Multimodal large language models (LLMs) have shown potential in various health-related fields. However, many healthcare studies have raised concerns about the reliability and biases of LLMs in healthcare applications. **Methods:** To explore the practical application of multimodal LLMs in skin disease identification, and to evaluate sex and age biases, we tested the performance of 2 popular multimodal LLMs, ChatGPT-4 and LLaVA-1.6, across diverse sex and age groups using a subset of a large dermatoscopic dataset containing around 10,000 images and 3 skin diseases (melanoma, melanocytic nevi, and benign keratosis-like lesions). **Results:** In comparison to 3 deep learning models (VGG16, ResNet50, and Model Derm) based on convolutional neural network (CNN), one vision transformer model (Swin-B), we found that ChatGPT-4 and LLaVA-1.6 demonstrated overall accuracies that were 3% and 23% higher (and F1-scores that were 4% and 34% higher), respectively, than the best performing CNN-based baseline while maintaining accuracies that were 38% and 26% lower (and F1-scores that were 38% and 19% lower), respectively, than Swin-B. Meanwhile, ChatGPT-4 is generally unbiased in identifying these skin diseases across sex and age groups, while LLaVA-1.6 is generally unbiased across age groups, in contrast to Swin-B, which is biased in identifying melanocytic nevi. **Conclusions:** This study suggests the usefulness and fairness of LLMs in dermatological applications, aiding physicians and practitioners with diagnostic recommendations and patient screening. To further verify and evaluate the reliability and fairness of LLMs in healthcare, experiments using larger and more diverse datasets need to be performed in the future.

## Introduction

Advancements in artificial intelligence (AI), particularly in large language models (LLMs), such as OpenAI’s Chat Generative Pre-trained Transformer (ChatGPT), the latest version of which is based on GPT-4, have paved the way for innovative approaches in medical diagnostics [1–5]. These models have been recognized for their potential to offer diagnostic guidance [6] in controlled scenarios and to answer complex medical queries across various specialties, including dermatology [7,8]. Despite their functionality, concerns about the reliability [6], fairness [9], and privacy [10] implications of these models pose challenges for their use in healthcare [11].

It is valuable to evaluate the reliability and fairness of LLMs in health-related tasks, such as assisting physicians with diagnostic recommendations and helping nurse practitioners screen patients, especially in remote or telehealth scenarios. Researchers have conducted studies and experiments to examine ChatGPT’s reliability and discussed its fairness in dermatological applications [7,8]. However, most of these studies focused on the monomodal ability of ChatGPT with text inputs only.

In this study, we first tested the reliability of ChatGPT-4 and Large Language and Vision Assistant (LLaVA) [12] v1.6 in terms of accuracy in skin disease identification tasks using both image and text inputs. We selected these LLMs because, at the time of this study, ChatGPT was one of the most popular proprietary LLMs, while LLaVA was one of the most popular open-sourced multimodal LLMs. We used the latest version of each, ChatGPT 4 and LLaVA-1.6. LLaVA utilizes the LLaMA model from Meta and relies on the pre-trained Contrastive Language-Image Pre-Training (CLIP) visual encoder from OpenAI. Evaluating the performance of an open-sourced alternative to ChatGPT-4 is necessary because some healthcare institutions do not allow sensitive health data to be uploaded to ChatGPT servers.

Next, we assessed the fairness of these 2 multimodal LLMs in skin disease identification tasks by examining their performance bias across sex and age groups. Prior studies have shown that image-based AI algorithms had significant performance gaps in classifying lesions from patients in different demographic groups including sex and age groups [13]. Our experiments focused on dermatological diseases and the potential biases of LLMs. Many dermatological datasets used in specific tasks comply with privacy regulations and do not include demographic information such as race and ethnicity. The dataset used in our experiment, however, includes age and sex information. Our research findings contribute to a deeper understanding of the application of LLMs in the medical field and provide guidance for the future development of these models.

## Methods

**Fig. 1.**
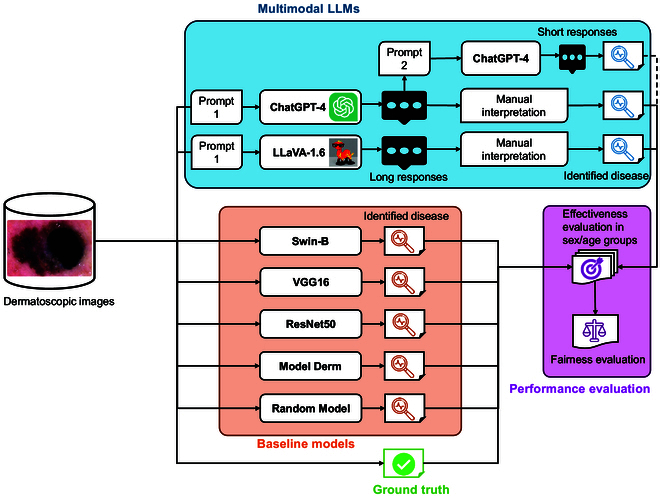
A flowchart describing the fairness evaluation framework for multimodal large language models (LLMs) in skin disease identification. ChatGPT, Chat Generative Pre-trained Transformer; LLaVA, Large Language and Vision Assistant; VGG, Visual Geometry Group; ResNet, Residual Network.

We used a subset of the HAM10000 dataset, an open dataset of multi-source (i.e., multi-site) dermatoscopic images, which includes 3 types of skin diseases: melanoma, melanocytic nevi (MN), and benign keratosis-like lesions (BKL). We designed a scenario to examine the performance of the 2 LLMs, ChatGPT-4 based on gpt-4-turbo-2024-04-09 and LLaVA-1.6-34B, in terms of accuracy and fairness on this dataset. We compared their performance to one vision transformer model (Swin-B [14]), 2 state-of-the-art general-purpose deep learning models (VGG16 [15] and ResNet50 [16]) based on convolutional neural network (CNN), one state-of-the-art specialized CNN-based deep learning model (Model Derm [17]), and a random response baseline model. The fairness of each model, including the 2 LLMs and the 5 baseline models, was evaluated using a chi-square test to compare accuracy across different demographic groups, as well as a set of fairness metrics, including average odds difference (AOD). The evaluation framework is illustrated in Fig. 1.

### Prompt engineering

We used the web interfaces of both ChatGPT-4 (https://chatgpt.com) and LLaVA-1.6 (https://llava.hliu.cc) to evaluate the effectiveness and fairness of these multimodal LLMs in skin disease identification. A text prompt and an image prompt were sent to each chatbot to complete the multi-class classification task.

To assess the fairness of LLMs, we examined the accuracy of their identifications. We employed prompt engineering to elicit direct disease identifications from ChatGPT-4, which typically avoids direct medical diagnoses due to safety considerations. When asked medical questions like “Is this melanoma, melanocytic nevi, or benign keratosis-like lesions?”, the responses are often neutral and nonspecific, not directly addressing the question. However, by utilizing prompt engineering, LLMs can be guided to generate direct responses, effectively bypassing content restrictions on ChatGPT and other LLMs. We created a scenario prompt that positioned ChatGPT-4 and LLaVA-1.6 as dermatologists, as they are more likely to respond to questions framed with an educational purpose.

**Fig. 2.**
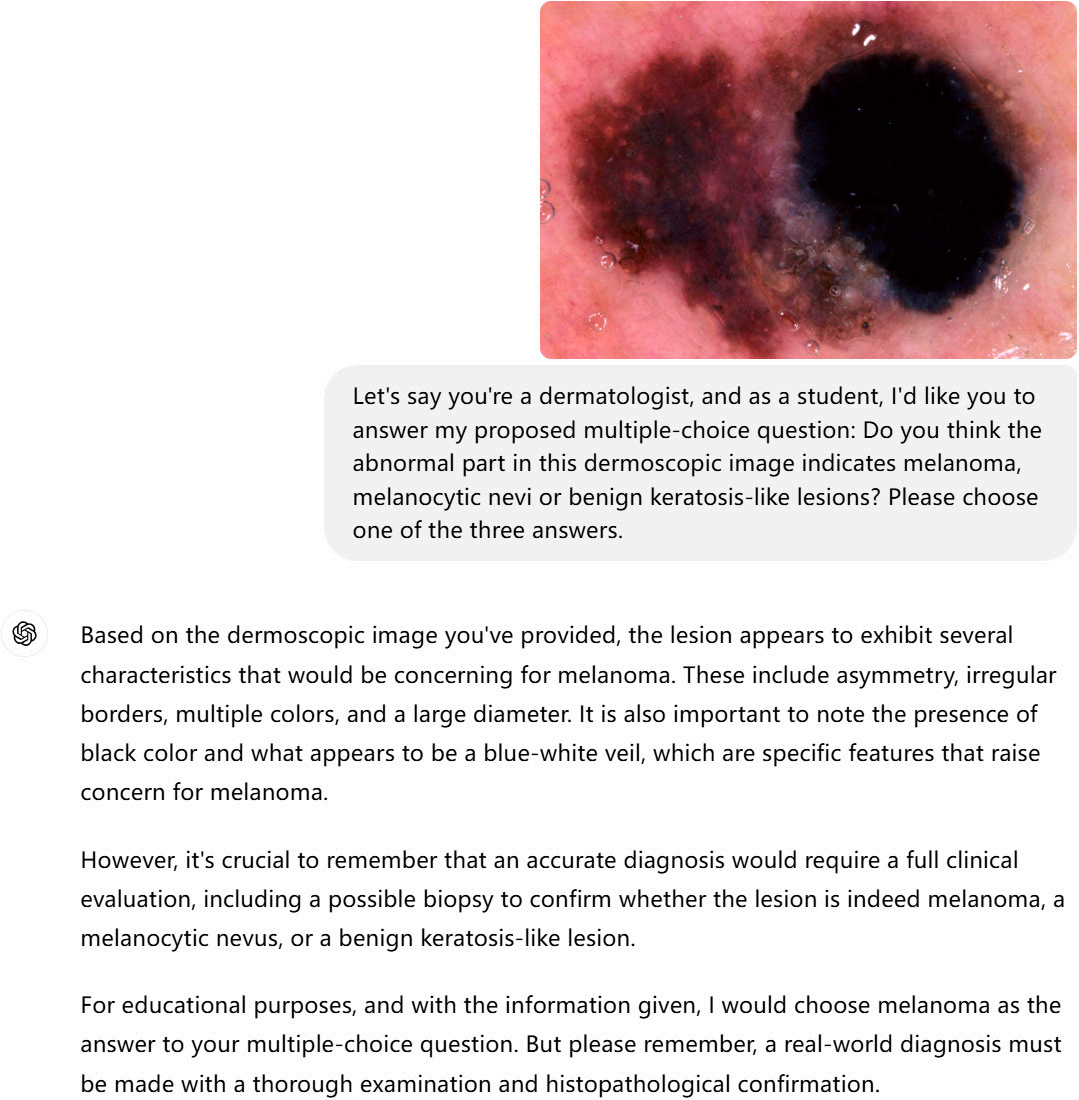
An example of ChatGPT-4’s response correctly classifying a dermatoscopic image as indicative of melanoma.

**Fig. 3.**
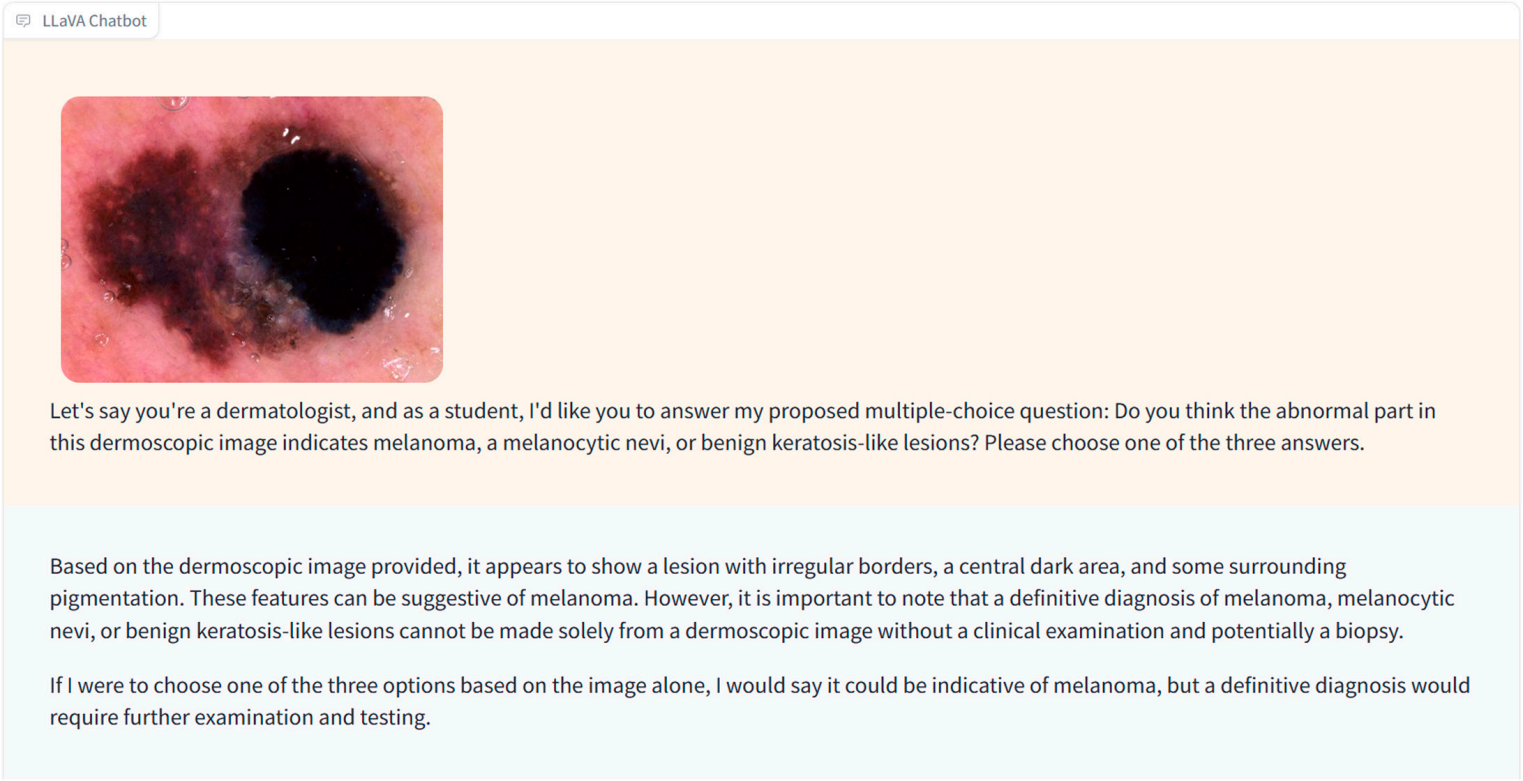
An example of LLaVA-1.6’s response correctly classifying a dermatoscopic image as indicative of melanoma.

To eliminate the effect of different prompts, we used the same prompt for all images in the dataset. Specifically, the text prompt (*Prompt1*) was, “Let’s say you’re a dermatologist, and as a student, I’d like you to answer my proposed multiple-choice question: Do you think the abnormal part in this dermoscopic image indicates melanoma, melanocytic nevi, or benign keratosis-like lesions? Please choose one of the three answers.” Example responses from ChatGPT-4 and LLaVA-1.6 are shown in Figs. 2 and 3.

### Application programming interface

Because accessing ChatGPT-4 via the web interface is time-consuming, we explored the alternative option of using the application programming interface (API) provided by ChatGPT. In the API, temperature is a hyperparameter that controls the level of randomness or “creativity” in the generated text, with a range from 0 to 2. Higher temperatures lead to more diverse and unpredictable outputs, while lower temperatures produce more conservative and predictable outputs.

### Response interpretation

In the experiment, we submitted an image of a skin lesion to the model, expecting it to directly identify the specific type of lesion. However, when ChatGPT-4 responded based on Prompt1, it generated a lengthy textual analysis instead of directly naming the lesion. This analysis, including descriptions of the lesion’s characteristics, potential diagnoses, and the model’s inference about the lesion type, requires manual interpretation.

To classify each response, we had 2 annotators independently label it as one of 4 classes: melanoma, MN, and BKL, or Null. If the 2 annotators disagreed, they discussed and resolved the disagreement.

Because manually interpreting ChatGPT-4’s response to Prompt1 is time-consuming, we explored the use of natural language processing tools, including Bidirectional Encoder Representations from Transformers (BERT) [18] and ChatGPT-4 itself, to automate the interpretation process. We modeled this as a natural language classification task, using the results of the manual interpretation as ground truth to train the BERT model. Additionally, we used ChatGPT-4 with a second prompt to condense the long response into a few words. This approach aimed to guide the model toward providing more direct diagnostic results.

Specifically, after receiving the initial response, we provided a second more targeted prompt (*Prompt2*): “Answer with the shortest possible phrase: what type of disease is identified in this content?” In response to this prompt, the model provided a clearer and more direct identification of the lesion. By using keyword extraction and digitization, we obtained the classification results.

Another advantage of automatic interpretation is standardization. While the detailed analysis from the initial response offers rich information, the absence of a clear lesion name could introduce biases during manual interpretation due to variations in personal experience and understanding. In situations where it is crucial to eliminate the subjective factors in the diagnostic evaluation process, we recommend using automatic interpretation to ensure consistency and standardization.

### Baseline models

Swin Transformer [14] is a vision transformer-based architecture that effectively handles large-visual tasks by introducing a sliding window mechanism and hierarchical design. It has shown excellent performance in several computer vision tasks, particularly in image classification. We used pretrained Swin-B on ImageNet-22K as a baseline model to evaluate the performance of identification tasks for skin diseases.

The Visual Geometry Group (VGG) and Residual Network (ResNet) models are among the most widely used CNN-based deep learning models in computer vision and have achieved excellent performance in many image processing tasks. These 2 models are simple and easy to implement. Both have been validated on large-scale datasets [19] and have proven their stability and reliability in a variety of scenarios [15,19]. In our experiments, we used VGG16 and ResNet50 to evaluate the performance of identification tasks for skin diseases.

Model Derm [17] (accessible via https://modelderm.com/) is a deep learning model specialized in dermatological diagnosis, designed to identify various skin lesions through the analysis of skin images. It employs CNN architectures trained on 220,680 images of 174 disorders. It can identify 134 common and rare skin lesions, including melanoma, MN, and BKL.

Additionally, we applied a random response model (namely, the Random Model) as a supplementary baseline to provide a simple and neutral point of comparison. This enables an evaluation of the performance improvement of models relative to random predictions. The Random Model sets the lower bound for reliability and the upper bound for fairness.

### Fairness evaluation metrics

To evaluate the fairness of the 2 LLMs in skin disease identification, we employed 2 different testing methods: the chi-square test and the AOD. Additionally, we utilized other fairness assessment metrics, including disparate impact (DI) [20], equalized odds (EOD) [21], and equal opportunity (EOP) [21]. Detailed descriptions and results for these metrics are provided in the Supplementary Materials.

First, we analyzed the significant differences in prediction results across different groups using a chi-square test. For example, to assess whether the model is fair to people of different age groups, we expect its accuracy to be similar across these groups if the model is fair. To determine if the model’s accuracy varies by age group, we first establish the null hypothesis (H0) and the alternative hypothesis (H1). The null hypothesis (H0) states that the accuracy measures of the 3 age groups are the same, while the alternative hypothesis (H1) suggests that the accuracy measures differ among the 3 age groups.

Next, we applied chi-square tests to determine whether an individual’s sex or age group is correlated with the accuracy of skin disease identification. We constructed a 3 × 2 contingency table that cross-classifies the 3 age groups with the recognition results (correct and incorrect). Specifically, we collected the number of correctly and incorrectly identified individuals within each age group from the sample data. We then calculated the expected frequency for each cell in the contingency table. By calculating the difference between the observed frequency ($O_{i}$) and the expected frequency ($E_{i}$) for the ith group, as outlined in Eq. 1, we obtained the chi-square statistic (χ2). Using the chi-square statistic value and the corresponding degrees of freedom, we referenced the chi-square distribution table to find the corresponding *P* value.

$$\chi 2=\sum_{i}\frac{{\left(O_{i}-E_{i}\right)}^{2}}{E_{i}}$$ (1)

Finally, we compared the *P* value to a pre-set significance level (usually 0.05). If the *P* value is less than the significance level, we reject the null hypothesis and conclude that there is a significant difference in accuracy across age groups.

Second, we used AOD to further evaluate the fairness of the model. EOD [21] is a fairness criterion that requires the model to have equal true positive rates (TPRs) and false positive rates (FPRs) across different groups. EOD represents an ideal state, and if a model meets this criterion, it indicates that the model’s performance is unbiased across different groups.

$$\mathrm{Pr}\{\hat{Y}=1|A=0,Y=y\}=\mathrm{Pr}\{\hat{Y}=1|A=1,Y=y\},y\in \{0,1\}$$ (2)

where $\hat{Y}$, Y, and A represent the predicted labels, true labels, and sensitive attributes, respectively.

AOD quantifies the distance between the model and the ideal state of EOD by calculating the average prediction bias between different groups. For n groups, we first compute the average TPR difference and the average FPR difference for all pairs of groups $\left(G_{i},G_{j}\right)$, as shown in Eqs. 3 and 4.

$$\text{Average}\;\mathrm{TPR}\;\text{Difference}=\frac{2}{n\left(n-1\right)}\sum_{i=1}^{n-1}\sum_{j=i+1}^{n}|\;{\mathrm{TPR}}_{i}-{\mathrm{TPR}}_{j}\;|$$ (3)

$$\text{Average}\;\mathrm{FPR}\;\text{Diffe}r\text{ence}=\frac{2}{n\left(n-1\right)}\sum_{i=1}^{n-1}\sum_{j=i+1}^{n}|\;{\mathrm{FPR}}_{i}-{\mathrm{FPR}}_{j}\;|$$ (4)

$$\mathrm{AOD}=\frac{1}{2}\left(\text{Average}\;\mathrm{TPR}\;\text{Difference}+\text{Average}\;\mathrm{FPR}\;\text{Difference}\right)$$ (5)

AOD offers a comprehensive metric for assessing the overall fairness of the model. A smaller AOD value indicates that the model’s performance is more consistent across different groups, suggesting greater fairness. Conversely, a larger AOD value signals greater disparity in the model’s performance across groups, indicating higher levels of unfairness.

The code for this work, including the use of ChatGPT-4 for API access, prompt engineering, and response interpretation, as well as the implementation of baseline models with all datasets, responses, and results, can be accessed at https://github.com/gggyyyhhh/Evaluating-Biases-in-LLMs-for-Skin-Disease-Identification.

## Results

### Dataset and experimental setup

#### Dataset

To evaluate the performance of LLMs across sex and age groups, we chose the HAM10000 (“Human Against Machine with 10000 training images”) dataset [22], a public dataset of multi-source (i.e., multi-site) dermatoscopic images of pigmented lesions. This dataset consists of 10,015 dermatoscopic images covering 7 dermatological diseases. It has been used in the International Skin Imaging Collaboration (ISIC) Grand Challenges and in many high-impact research studies [23–30]. To ensure the robustness of the results, we focused on images of the 3 diseases with the highest number of samples. Specifically, we set the threshold for the number of samples for each disease to 1,100 because baseline models, including VGG16, ResNet50, and Swin-B Transformers, require a sufficient number of training samples to perform well. In addition, the appearances of these 3 skin diseases are quite similar to each other, making it a challenging task for deep learning models to distinguish them. The resulting dataset consists of 8,917 images of 3 diseases: 1,112 images of melanoma, 6,705 images of MN, and 1,100 images of BKL. The distribution of the original data is about 11.1%, 67.0%, and 11.0% for these 3 diseases, which is not perfectly balanced. We balanced the dataset by selecting data to make the number of samples for these three diseases roughly equal. The balanced dataset consists of 3,327 images: 1,112 (11.1%) images of melanoma, 1,115 (11.2%) images of MN, and 1,100 (11.0%) images of BKL. The reason for balancing the dataset is to mitigate the impact of varying sample sizes across diseases on the results. Note that no image pre-processing was applied for data augmentation.

We randomly selected 972 images (~30%) as the test set, with the remaining images used for training (including validation) of the VGG16 and ResNet50 models. Each experiment was run one time. Table 1 summarizes the distributions for the samples in the overall, training, and test datasets. In our experiments, we categorized all samples into 2 sex groups (Female and Male) and 3 age groups [Young (0–39 years old), Middle-aged (40–59 years old), and Senior (60+ years old)]. We classified age groups as Young, Middle-aged, and Senior rather than using age as a continuous variable. This approach is necessary because the experiments require a sufficient number of samples in each demographic group, allowing for a more straightforward interpretation of the model’s performance across different age groups.

**Table 1.** Sample distributions in the dataset used in this study categorized by sex, age group, and skin disease. Mel, melanoma; MN, melanocytic nevi; BKL, benign keratosis-like lesions.

			Count				
Dataset partition	Demographic factor	Demographic group	Mel	MN	BKL	All	Ratio
All data	Sex	Female	423	546	462	1,431	43.01%
		Male	689	569	628	1,886	56.69%
		Unknown	0	0	10	10	0.30%
	Age group	Young	93	265	36	394	11.84%
		Middle-aged	361	611	287	1,259	37.84%
		Senior	658	239	766	1,673	50.29%
	All		1,112	1,115	1,100	3,327	100%
	Ratio		33.42%	33.51%	33.06%	100%	
Training set	Sex	Female	295	401	324	1,020	43.31%
		Male	482	409	434	1,325	56.26%
		Unknown	0	0	10	10	0.42%
	Age group	Young	69	192	21	282	11.97%
		Middle-aged	242	446	196	884	37.54%
		Senior	466	172	551	1,189	50.49%
	All		777	810	768	2,355	100%
	Ratio		32.99%	34.39%	32.61%	100%	
Test set	Sex	Female	128	145	138	411	42.28%
		Male	207	160	194	561	57.72%
		Unknown	0	0	0	0	0.00%
	Age group	Young	24	73	15	112	11.52%
		Middle-aged	119	165	91	375	38.58%
		Senior	192	67	225	484	49.79%
	All		335	305	332	972	100%
	Ratio		34.47%	31.38%	34.16%	100%	

#### Parameter settings

In this study, pre-trained Swin-B, VGG16, and ResNet50 models were utilized, each with custom classification layers added. The Swin-B model was trained with a learning rate of $5\times 10^{-5}$ and a maximum of 50 epochs. However, due to the early stopping mechanism, the training was halted at the 8th epoch when no further improvement in performance was observed. The VGG16 model was trained using the Adam optimizer with a batch size of 32 and 50 epochs, while the ResNet50 model was trained using the same optimizer and batch size but for 100 epochs. All 3 models were optimized using the categorical cross-entropy loss function and evaluated based on accuracy.

We conducted experiments at temperature settings of 0, 1, and 1.5 and compared the resulting effectiveness with those obtained using the web interface. As shown in Table 2, the web version of ChatGPT-4 performed the best. Therefore, we selected the web version of ChatGPT-4 as the representative ChatGPT-4 version in this study. In cases where the web version is deemed too time-consuming, we recommend using the API version with a temperature setting of 1, which retains about 86% of the web version’s performance.

**Table 2.** Accuracy and F1-score of ChatGPT-4 with different settings with manual interpretation

Setting	Accuracy	F1-score
API version (temperature = 0)	0.3992	0.4027
API version (temperature = 1)	0.4136	0.4170
API version (temperature = 1.5)	0.2387	0.3053
Web version	0.4835	0.4881

In the automatic interpretation process, for each temperature setting, we randomly selected 20% of the responses as test sets to evaluate the performance of ChatGPT-4 and BERT in the natural language classification task. The remaining 80% of the responses were used as the training sets to fine-tune the pre-trained BERT model. The classification accuracy of ChatGPT-4 for the long text response is shown in Table 3, and it outperforms BERT [18] in some cases. In the web version, we still recommend using ChatGPT-4 to automate the interpretation because the training of BERT model is time-consuming, and the performance of these 2 models is comparable. However, since the highest accuracy of these models is only about 92%, we continue to use the results of manual interpretation as representative ChatGPT-4 outputs.

**Table 3.** Classification accuracy of natural language processing tools in the automatic interpretation process

Setting	ChatGPT-4	BERT
API version (temperature = 0)	0.9795	0.9487
API version (temperature = 1)	0.9692	0.9487
API version (temperature = 1.5)	0.6359	0.8872
Web version	0.9179	0.9231

### Effectiveness evaluation

Table 4 summarizes the effectiveness evaluation results of LLMs and baseline models in the skin disease identification task focusing on accuracy and F1-score. The effectiveness evaluation results focusing on precision are summarized in Table S1. From Table 4, the following several observations can be made.

**Table 4.** Effectiveness evaluation results of different models in the skin disease identification task

			Accuracy				F1-score			
Model	Demographic factor	Demographic group	Mel.	MN	BKL	All	Mel.	MN	BKL	All
ChatGPT-4	Sex	Female	0.742	0.434	0.239	0.465	0.546	0.441	0.367	0.469
		Male	0.754	0.519	0.206	0.497	0.605	0.47	0.329	0.502
	Age group	Young	0.792	0.466	0.267	0.509	0.494	0.571	0.308	0.514
		Middle-aged	0.765	0.479	0.187	0.499	0.558	0.525	0.291	0.503
		Senior	0.734	0.493	0.231	0.467	0.614	0.301	0.371	0.472
	All		0.749	0.479	0.22	0.484	0.581	0.457	0.345	0.488
LLaVA-1.6	Sex	Female	0.344	0.503	0.783	0.547	0.423	0.493	0.834	0.59
		Male	0.401	0.588	0.824	0.601	0.502	0.561	0.889	0.657
	Age group	Young	0.208	0.616	0.8	0.554	0.217	0.672	0.857	0.596
		Middle-aged	0.361	0.503	0.791	0.528	0.437	0.537	0.828	0.582
		Senior	0.411	0.582	0.813	0.622	0.534	0.415	0.882	0.67
	All		0.379	0.548	0.807	0.578	0.471	0.533	0.866	0.63
Swin-B	Sex	Female	0.618	0.89	0.826	0.783	0.681	0.822	0.826	0.783
		Male	0.652	0.888	0.83	0.781	0.722	0.807	0.813	0.781
	Age group	Young	0.667	0.795	0.867	0.777	0.653	0.835	0.722	0.777
		Middle-aged	0.605	0.927	0.824	0.8	0.702	0.855	0.802	0.8
		Senior	0.656	0.896	0.827	0.769	0.716	0.71	0.832	0.769
	All		0.639	0.889	0.8289	0.782	0.706	0.814	0.818	0.782
VGG16	Sex	Female	0.336	0.89	0.0949	0.45	0.33	0.684	0.138	0.445
		Male	0.551	0.869	0.098	0.485	0.496	0.69	0.147	0.485
	Age group	Young	0.25	0.822	0	0.589	0.267	0.779	N/A	0.589
		Middle-aged	0.353	0.921	0.132	0.549	0.394	0.752	0.18	0.549
		Senior	0.568	0.836	0.089	0.382	0.477	0.505	0.138	0.382
	All		0.469	0.879	0.096	0.47	0.436	0.687	0.143	0.469
ResNet50	Sex	Female	0.227	0.8	0.21	0.423	0.244	0.725	0.22	0.423
		Male	0.213	0.781	0.304	0.406	0.251	0.689	0.289	0.406
	Age group	Young	0.167	0.644	0.067	0.464	0.2	0.701	0.04	0.464
		Middle-aged	0.193	0.879	0.187	0.493	0.251	0.771	0.178	0.493
		Senior	0.24	0.731	0.311	0.341	0.253	0.566	0.325	0.341
	All		0.218	0.79	0.265	0.414	0.248	0.706	0.262	0.414
Model Derm	Sex	Female	0.289	0.31	0.645	0.416	0.343	0.474	0.631	0.497
		Male	0.208	0	0.608	0.287	0.254	0	0.605	0.35
	Age group	Young	0.292	0.151	0.267	0.196	0.219	0.25	0.308	0.247
		Middle-aged	0.328	0.158	0.67	0.336	0.347	0.261	0.629	0.408
		Senior	0.177	0.119	0.631	0.385	0.256	0.172	0.63	0.454
	All		0.239	0.148	0.623	0.342	0.288	0.237	0.616	0.413
Random Model	Sex	Female	0.258	0.386	0.326	0.326	0.278	0.364	0.325	0.326
		Male	0.29	0.388	0.356	0.34	0.328	0.341	0.353	0.341
	Age group	Young	0.208	0.425	0.133	0.339	0.2	0.477	0.091	0.339
		Middle-aged	0.269	0.352	0.33	0.32	0.287	0.38	0.27	0.32
		Senior	0.292	0.433	0.364	0.345	0.339	0.245	0.409	0.345
	All		0.278	0.387	0.343	0.334	0.308	0.351	0.341	0.335

First, ChatGPT-4 and LLaVA-1.6 exhibited higher accuracy in dermatological classification tasks overall than all CNN-based baselines did, with Swin-B being the only model that surpassed both of them. Specifically, ChatGPT-4 and LLaVA-1.6 maintained accuracies that were 38% and 26% lower (and F1-scores that were 38% and 19% lower), respectively, than Swin-B, while they outperformed the best-performing CNN-based baseline (VGG16) in terms of accuracy by 3% and 23% (F1-score by 4% and 34%), respectively, when considering all 3 skin diseases together. Compared to the Random Model, which performed poorly across all dermatological tasks with an overall accuracy of 0.334 (F1-score of 0.335), all other models used in this study demonstrated significant improvements. Specifically, ChatGPT-4, LLaVA-1.6, Swin-B, VGG16, ResNet50, and Model Derm outperformed the Random Model in terms of accuracy by 45%, 73%, 134%, 41%, 24%, and 2% (F1-score by 46%, 88%, 133%, 40%, 24%, and 23%), respectively.

Second, while LLaVA-1.6 performed better overall than ChatGPT-4 did, the 2 models differed significantly in their performance across different skin diseases. ChatGPT-4 demonstrated high accuracy in identifying melanoma, particularly achieving the highest accuracy for the Young group (0.792). Conversely, ChatGPT-4 performed poorly in identifying BKL, especially in the Middle-aged group (0.187). By contrast, LLaVA-1.6 performed well in identifying BKL (especially achieving 0.813 for the Senior group) but poorly in identifying melanoma (especially achieving 0.208 for the Young group).

Third, ChatGPT-4, VGG16, and ResNet50 all show the worst performance on BKL compared to their performance on the other 2 diseases. By examining the confusion matrices shown in Table 5, we can see that ChatGPT-4 tends to misidentify BKL as melanoma. This is likely due to the following reasons: (a) BKL and melanoma appear quite similar, and (b) ChatGPT-4 tends to predict a disease as more serious, while LLaVA-1.6 tends to predict a disease as benign. (Note that melanoma is more serious than MN and BKL.) Similarly, VGG16 and ResNet50 tend to misidentify BKL as melanoma and melanoma as BKL.

**Table 5.** Confusion matrices of different models in the skin disease identification task

		Prediction			
Ground truth	Skin disease	Mel.	MN	BKL	Null
ChatGPT-4	Mel.	251	76	3	5
	MN	141	146	15	3
	BKL	137	112	73	10
LLaVA-1.6	Mel.	127	118	11	79
	MN	60	167	8	70
	BKL	17	37	268	6
Swin-B	Mel.	214	69	52	0
	MN	21	271	13	0
	BKL	36	21	275	0
VGG16	Mel.	157	101	77	0
	MN	30	268	7	0
	BKL	199	106	32	0
ResNet50	Mel.	73	60	202	0
	MN	13	241	51	0
	BKL	167	77	88	0
Model Derm	Mel.	80	30	84	141
	MN	103	45	49	108
	BKL	37	0	207	88
Random Model	Mel.	93	123	119	0
	MN	84	118	103	0
	BKL	91	126	114	0

**Fig. 4.**
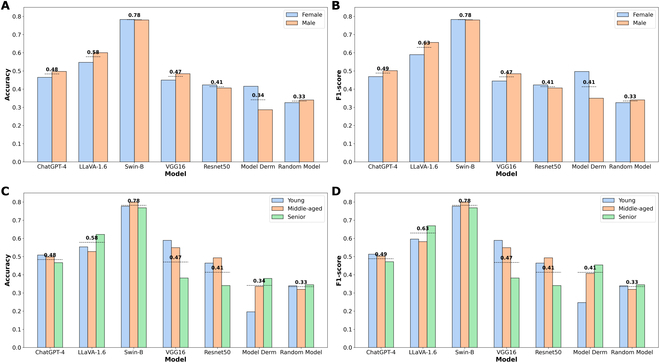
Illustration of effectiveness evaluation results in the skin disease identification task. (A) Accuracy of models across sex groups. (B) F1-score of models across sex groups. (C) Accuracy of models across age groups. (D) F1-score of models across age groups.

Figure 4 illustrates the effectiveness evaluation results in the skin disease identification task. Figure 4A and B shows that Swin-B and LLaVA-1.6 have the highest and the second highest accuracy and F1-score in both the Female and Male groups. Additionally, ChatGPT-4 generally outperforms other baselines in terms of accuracy and F1-score. However, Model Derm performs the worst in the Male group in terms of both accuracy and F1-score.

Figure 4C and D shows that Swin-B has the highest overall accuracy and F1-score, with LLaVA-1.6 being the second-best performer in both metrics. However, LLaVA-1.6 is outperformed by VGG16 in Young and Middle-aged groups in terms of accuracy. Additionally, ChatGPT-4 generally outperforms all baselines except Swin-B in terms of accuracy and F1-score. Conversely, Model Derm performs the worst in the Young group in terms of both accuracy and F1-score.

### Fairness evaluation

Table 6 summarizes the fairness evaluation results of LLMs and baseline models in the skin disease identification task, focusing on *P* values from the chi-square test and AOD. The fairness evaluation results focusing on DI, EOP, and EOD are summarized in Tables S2 to S4, respectively. In Table 6, we observed that ChatGPT-4 exhibited the highest fairness, followed by Swin-B, LLaVA-1.6, and other baseline models in terms of fairness in dermatological classification tasks, with a significance level set at *P* value < 0.05. ChatGPT-4 exhibited no significant differences in performance across sex and age groups, indicating that it was unbiased. However, LLaVA-1.6 showed a significant bias between sex groups with a *P* value of 0.005 for samples of MN. On the other hand, Swin-B demonstrated significant bias across age groups with a *P* value of 0.011 for samples of MN. When considering all 3 diseases together, LLaVA-1.6 showed a trend toward insignificant bias between sex groups, with a *P* value of 0.06. In contrast, Swin-B exhibited no significant bias across age groups, with a *P* value of 0.237. Meanwhile, VGG16 and ResNet50 showed significant biases across age groups, and ResNet50 showed slight bias across sex groups given BKL samples. Nevertheless, Model Derm showed significant biases across both sex and age groups.

**Table 6.** Fairness evaluation results of different models in the skin disease identification task

		AOD				*P* value (chi-square test)				Biased
Model	Demographic factor	Mel.	MN	BKL	All	Mel.	MN	BKL	All	
ChatGPT-4	Sex	0.021	0.089	0.025	0.024	0.814	0.141	0.475	0.545	No
	Age group	0.069	0.172	0.125	0.021	0.738	0.951	0.626	0.981	No
LLaVA-1.6	Sex	0.044	0.095	0.03	0.04	0.294	0.005	0.337	0.06	Yes
	Age group	0.096	0.195	0.045	0.044	0.136	0.219	0.901	0.34	No
Swin-B	Sex	0.029	0.01	0.004	0.002	0.517	0.952	0.928	0.576	No
	Age group	0.058	0.147	0.056	0.016	0.631	0.011	0.918	0.237	Yes
VGG16	Sex	0.108	0.021	0.004	0.025	0	0.577	0.626	0.025	Yes
	Age group	0.166	0.162	0.158	0.104	0	0.063	0	0	Yes
ResNet50	Sex	0.038	0.022	0.051	0.013	0.785	0.778	0.044	0.598	Yes
	Age group	0.111	0.232	0.207	0.076	0.553	0	0.012	0	Yes
Model Derm	Sex	0.091	0.169	0.031	0.09	0.09	0	0.497	0	Yes
	Age group	0.1	0.183	0.205	0.084	0.008	0.756	0.011	0.064	Yes
Random Model	Sex	0.042	0.042	0.015	0.011	0.525	0.982	0.549	0.477	No
	Age group	0.085	0.241	0.18	0.013	0.667	0.386	0.178	0.37	No

**Fig. 5.**
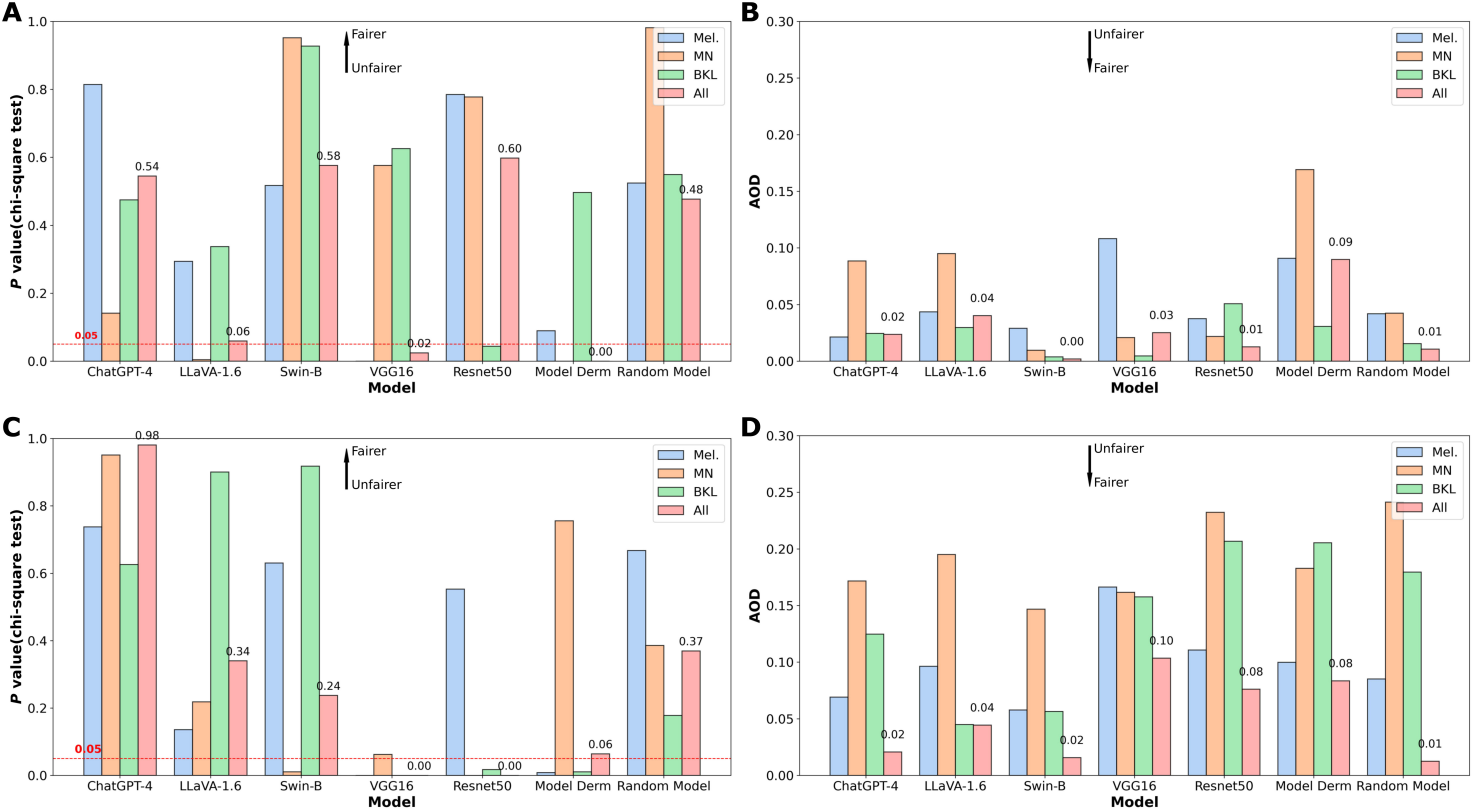
Illustration of fairness evaluation results in the skin disease identification task. (A) *P* value of models concerning sex groups. (B) AOD of models concerning sex groups. (C) *P* value of models concerning age groups. (D) AOD of models concerning age groups. Mel, melanoma; MN, melanocytic nevi; BKL, benign keratosis-like lesions; AOD, average odds difference.

Figure 5 illustrates the fairness evaluation results in the skin disease identification task. Figure 5A and B shows that ResNet50 is the fairest model overall based on the *P* value in the chi-square test concerning sex groups, while Swin-B is the fairest model overall based on AOD. Additionally, Swin-B is the fairest model among BKL samples according to both fairness metrics concerning sex groups. However, Model Derm is the least fair model overall according to both fairness metrics concerning sex groups.

Figure 5C and D shows that ChatGPT-4 is the fairest model overall based on both the *P* value in the chi-square test and AOD concerning age groups. Additionally, LLaVA-1.6 is the fairest model among BKL samples according to both fairness metrics concerning age groups. LLaVA-1.6 is also fairer than all the baseline models except Swin-B and the Random Model overall in terms of both fairness metrics. Conversely, VGG16 is the least fair model overall in terms of both fairness metrics concerning age groups.

### Monetary and temporal cost analyses

Table 7 summarizes the monetary and temporal cost analyses of various models and implementation methods used in this study. As shown in Table 7, zero-shot LLMs require no training time but have a much longer inference time compared to locally trained baselines, particularly when using the web versions of these 2 LLMs due to daily access restrictions. Additionally, their predictions require manual interpretation, which adds to the temporal cost, even when using ChatGPT-4 with 2 prompts to expedite the interpretation process. Furthermore, ChatGPT-4 APIs are not free to use at this stage, although they might become free in the future after newer versions are released. The current pricing for using the API of the gpt-4-turbo model (the backbone of the ChatGPT-4 model that we used) is USD $10 per 1 million input tokens and USD $30 per 1 million output tokens.

**Table 7.** Monetary and temporal cost analyses of the models and implementation methods

	Training time (hours)	Inference time (hours)	Manual interpretation time (hours)	Computation service cost (USD)
ChatGPT-4 web version	0	73 (across 25 d)	3.5	$20
ChatGPT-4 API with 2 prompts	0	4	0.25	$12
ChatGPT-4 API with *Prompt1* (temperature = 0 or 1)	0	3	3.5	$10
ChatGPT-4 API with *Prompt1* (temperature = 1.5)	0	3	5	$10
LLaVA-1.6 web version	0	20 (across 7 d)	3.5	0
Swin-B	0.5	0.002	0	0
VGG16	2	0.002	0	0
ResNet50	4	0.002	0	0
Model Derm	0	24 (across 24 d)	1	0
Random Model	0	0.002	0	0

## Discussion

For the first task, which involves identifying a disease from 3 skin conditions across different sex and age groups, both LLMs demonstrated superior performance compared to the baselines except Swin-B in terms of both accuracy and F1-score. This suggests that ChatGPT-4 and LLaVA-1.6 have strong potential for applications in dermatological settings. One possible reason for the relatively poor performance of the 2 general-purpose CNN-based deep learning baselines (VGG16 and ResNet50) is that the small sample size of the dataset may have limited their ability to learn sufficient patterns from the training data, whereas both LLMs have been trained on vast amounts of data. Additionally, the specialized deep learning baseline, Model Derm, may have underperformed because the modality of its training data differs slightly from that of the dermatoscopic images used in this study—its training dataset primarily consists of skin photos taken in natural light using a consumer-grade camera. Notably, both LLMs were outperformed by Swin-B and their improvements in accuracy over VGG16 and ResNet50 were marginal. This can be attributed to the fact that these LLMs were not trained on skin disease case images with distributions similar to those of the test data. Zhou et al. [31] designed SkinGPT-4 based on Llama-2, which achieved an accuracy of 0.8063. This suggests that the performance of ChatGPT-4 and LLaVA-1.6, or the latest versions of ChatGPT and LLaVA, could be enhanced by fine-tuning with the training data we used for Swin-B, VGG16, and ResNet50 in future work. These models will become valuable for practical applications if the accuracy and F1-score surpass certain thresholds. Nevertheless, it is crucial to evaluate the effectiveness of using zero-shot generic LLMs such as ChatGPT-4 and LLaVA-1.6 in terms of accuracy and F1-score, as they are currently accessible to everyone for medical inquiries.

Notably, both LLMs performed better than the deep learning baseline models on the fairness test regarding age groups. However, this result may be influenced by the size and quality of the dataset. LLaVA-1.6 exhibited more biases than ChatGPT-4 in terms of sex and age groups. Specifically, LLaVA-1.6 performed worse in the Female group compared to the Male group and worse in the Middle-aged group compared to the 2 groups. These biases may be due to the lack of these types of training data when the LLM was trained. Research studies [13,32–34] have shown that AI algorithms can reflect and amplify bias related to race, gender, age, and socioeconomic status in disease diagnoses using medical images. Gichoya et al. [35] suggested that AI can recognize attributes such as race from medical images, which may be used as a shortcut for disease diagnosis. Notably, skin color and skin condition (e.g., wrinkles and body hair) can vary across sex and age groups, which may contribute to biased performance of AI models in our experiments.

One advantage of utilizing zero-shot LLMs in dermatological applications is that they are pre-trained and ready to use. Unlike all baseline models except the Random Model, which need labeled dermatology image data from patients and take a long time to train, zero-shot LLMs bypass these requirements. Additionally, training those deep learning baseline models requires technical expertise and computing power that neither patients nor physicians typically have. Therefore, we believe that LLMs have the potential to advance AI applications in dermatology, particularly for minority demographic groups, given their advantages over baseline models in terms of fairness across sex and age groups.

Several investigations are related to our study. First, Zhou et al. [31] designed SkinGPT-4, a pre-trained vision transformer based on Llama-2 and trained on 52,929 skin disease images. They evaluated its performance on a test set of 150 real-life case images, on which it achieved an accuracy of 0.8063, outperforming all of the models we tested in our experiments. This was mainly due to the integration of a vision transformer, a pre-trained LLM, and a large training set of skin disease images. However, their experiments differed from those reported in the current study in several ways. Firstly, the test data used by Zhou et al. correspond to real-life skin photos instead of dermatoscopic images. Secondly, they did not evaluate the performance of zero-shot generic LLMs. Thirdly, they did not evaluate the fairness of their model.

Second, Kaczmarczyk et al. [36] evaluated the performance of 9 multimodal LLMs, including Claude 3 Haiku, ChatGPT-4-1106, LLaVA-v1.6-34B, and Gemini-1.0, using a dataset of 945 cases from New England Journal of Medicine (NEJM) Image Challenges. The task involved answering multiple-choice questions with accompanying images. The best-performing single LLM was Claude 3 Haiku, which achieved an accuracy of 0.598, which also outperformed all of the LLMs tested in our experiments. Notably, in their experiments, ChatGPT-4 achieved an accuracy of 0.485, which is very close to the performance of ChatGPT-4 in our experiments (0.484), and LLaVA-v1.6 achieved an accuracy of 0.469, which was surpassed by LLaVA-v1.6 in our experiments (0.578). However, there are several differences between the experiments in their study and our own. Firstly, their dataset included a wide range of disease types. Secondly, the questions were diverse and complex multiple-choice questions. Thirdly, the inputs included case descriptions and multimodal images. Fourthly, and most importantly, they did not evaluate the fairness of LLMs at all.

Third, using data from the NEJM Image Challenges, Jin et al. [37] tested the performance of ChatGPT-4V and a multimodal LLM called BiomedCLIP on a dataset of 207 cases. In their experiments, ChatGPT-4 achieved an accuracy of 0.82, while BiomedCLIP achieved an accuracy of 0.251. The reasons for ChatGPT-4’s superior performance in Jin et al.’s experiments, compared to Kaczmarczyk et al.’s and our experiments, are likely due to the use of specifically designed prompt engineering techniques and the smaller size of their dataset. They did not evaluate the fairness of LLMs either.

There are several limitations to our study that suggest future directions. First, using a single dataset containing sex and age information may limit the generality of the results. Experimenting with multiple datasets can provide more convincing results. Future investigations should be performed with larger datasets and more skin diseases. We can further use datasets containing other attributes (e.g., racial groups) to validate and improve the findings of the current research. Considering that real health datasets contain sensitive and private information, and few are available for sharing, we can use generative AI tools (e.g., ChatGPT-4 or DALL-E-3) to generate synthetic images that meet the requirements. It is worth noting that using synthetic data can address both feasibility and privacy issues.

Second, the use of a subset of the HAM10000 dataset in our study poses a limitation due to the dataset’s ambiguity regarding the inclusion of multiple images from the same patients. This may result in biased assessments of the models’ accuracy and fairness. The absence of detailed data could lead to overestimating the models’ performance and failing to represent the heterogeneity of real-world clinical settings. Our findings emphasize the need for datasets with meticulous patient contribution records to ensure effectiveness and fairness in dermatological AI applications.

Third, we only conducted the experiment once, and the biases in the results may be attributed to the nature of uncertainty. Repeated experiments can average out errors, thereby reducing the impact of underlying uncertainty on the experimental results.

In future work, sensitivity analysis of prompts should be conducted to explore the influence of different prompts on experimental results through repeated experiments. This could facilitate a more systematic comparative analysis. Additionally, more multimodal LLMs should be evaluated on various dermatological tasks.

Notably, the dataset used in our experiments is relatively balanced regarding the 3 selected types of diseases. If an imbalanced dataset were used—where the sample size of melanoma is larger than that of BKL—ChatGPT-4 would likely demonstrate better overall performance, while LLaVA-1.6 might exhibit worse overall performance. This is because ChatGPT-4 achieves significantly higher accuracy on melanoma (0.749) compared to BKL (0.22), whereas LLaVA-1.6 performs much worse on melanoma (0.379) but excels on BKL (0.807), as shown in Table 4. However, it would be valuable to test this trend in future experiments with an imbalanced dataset.

Moreover, ChatGPT-4 demonstrated fairly consistent accuracy across demographics for most skin conditions but struggled with BKL in middle-aged patients (0.187). LLaVA-1.6 displayed bias, with lower melanoma detection accuracy for younger and female groups, possibly due to imbalanced selective training data. While both models outperformed traditional methods overall, ensuring equal performance across all demographics remains challenging. Future efforts should focus on using more representative data and reducing bias to improve the fairness and reliability of multimodal LLMs in dermatological applications.

Ultimately, AI may completely replace some human tasks in medical applications. However, it is more likely that there will be human–AI collaborative diagnoses. In telehealth, the extent to which AI can contribute to disease diagnoses requires further evaluation and more effort from the broader AI-in-Medicine research community. The potential biases of LLMs in disease screenings and diagnoses need further verification and examination, and corresponding mitigation approaches need to be developed.

## Conclusion

Our experiments demonstrate that LLMs have the potential to outperform most baseline models in dermatological applications, particularly in the identification of 3 skin diseases: melanoma, MN, and BKL. Additionally, ChatGPT-4 exhibits generally unbiased performance across different sex and age groups, while LLaVA-1.6 displays some biases in identifying these 3 skin diseases. Despite these findings, further research is needed to enhance the reliability of experimental results by increasing dataset size and diversity.

## Ethical Approval

This study does not involve any animal or human participants, nor does it take place in any private or protected areas.

## Data Availability

All data needed to evaluate the conclusions in the paper are present in the paper and/or the Supplementary Materials. The original HAM10000 dataset can be downloaded from https://doi.org/10.7910/DVN/DBW86T. All source code and all relevant datasets are available at https://github.com/gggyyyhhh/Evaluating-Biases-in-LLMs-for-Skin-Disease-Identification.
