# Supplementary material for: Evaluating Sex and Age Biases in Multimodal Large Language Models for Skin Disease Identification from Dermatoscopic Images
Source: Health Data Sci. 2025 Apr 1;5:0256. doi: 10.34133/hds.0256 (PMC11961048; doi:10.34133/hds.0256)
Supplement: Supplementary 1 — Tables S1 to S4 Supplementary Text [file hds.0256.f1.docx]

# **Supplementary Materials**

## **S1. Supplementary Tables**

**Table S1.** Effectiveness evaluation results of different models in the skin disease identification task in terms of precision. Mel: melanoma; MN: melanocytic nevi; BKL: benign keratosis-like lesions.

|  | | | Precision | | | |
| --- | --- | --- | --- | --- | --- | --- |
|  |  |  | Mel. | MN | BKL | All |
| ChatGPT-4 | Sex | Female | 0.432 | 0.447 | 0.786 | 0.474 |
|  |  | Male | 0.505 | 0.43 | 0.816 | 0.506 |
|  | Age group | Young | 0.358 | 0.739 | 0.364 | 0.518 |
|  |  | Middle-aged | 0.44 | 0.581 | 0.654 | 0.507 |
|  |  | Senior | 0.528 | 0.217 | 0.945 | 0.477 |
|  | All | | 0.474 | 0.437 | 0.802 | 0.493 |
| LLaVA-1.6 | Sex | Female | 0.55 | 0.483 | 0.893 | 0.639 |
|  |  | Male | 0.669 | 0.537 | 0.964 | 0.725 |
|  | Age group | Young | 0.227 | 0.738 | 0.923 | 0.646 |
|  |  | Middle-aged | 0.551 | 0.576 | 0.867 | 0.649 |
|  |  | Senior | 0.76 | 0.322 | 0.963 | 0.725 |
|  | All | | 0.623 | 0.519 | 0.934 | 0.691 |
| Swin-B | Sex | Female | 0.76 | 0.763 | 0.826 | 0.783 |
|  |  | Male | 0.808 | 0.74 | 0.797 | 0.781 |
|  | Age group | Young | 0.64 | 0.879 | 0.619 | 0.777 |
|  |  | Middle-aged | 0.837 | 0.793 | 0.781 | 0.8 |
|  |  | Senior | 0.788 | 0.588 | 0.839 | 0.769 |
|  | All | | 0.79 | 0.751 | 0.809 | 0.782 |
| VGG16 | Sex | Female | 0.323 | 0.556 | 0.255 | 0.445 |
|  |  | Male | 0.451 | 0.572 | 0.292 | 0.485 |
|  | Age group | Young | 0.286 | 0.741 | 0 | 0.589 |
|  |  | Middle-aged | 0.447 | 0.636 | 0.286 | 0.549 |
|  |  | Senior | 0.411 | 0.361 | 0.313 | 0.382 |
|  | All | | 0.407 | 0.564 | 0.276 | 0.468 |
| ResNet50 | Sex | Female | 0.264 | 0.663 | 0.23 | 0.423 |
|  |  | Male | 0.308 | 0.616 | 0.274 | 0.406 |
|  | Age group | Young | 0.25 | 0.77 | 0.029 | 0.464 |
|  |  | Middle-aged | 0.359 | 0.687 | 0.17 | 0.493 |
|  |  | Senior | 0.267 | 0.462 | 0.34 | 0.341 |
|  | All | | 0.289 | 0.638 | 0.258 | 0.414 |
| Model Derm | Sex | Female | 0.42 | 1 | 0.618 | 0.617 |
|  |  | Male | 0.326 | 0 | 0.602 | 0.45 |
|  | Age group | Young | 0.175 | 0.733 | 0.364 | 0.333 |
|  |  | Middle-aged | 0.368 | 0.765 | 0.592 | 0.519 |
|  |  | Senior | 0.459 | 0.308 | 0.628 | 0.564 |
|  | All | | 0.364 | 0.6 | 0.609 | 0.523 |
| Random Model | Sex | Female | 0.303 | 0.344 | 0.324 | 0.326 |
|  |  | Male | 0.377 | 0.304 | 0.35 | 0.341 |
|  | Age group | Young | 0.192 | 0.544 | 0.069 | 0.339 |
|  |  | Middle-aged | 0.308 | 0.414 | 0.229 | 0.32 |
|  |  | Senior | 0.406 | 0.171 | 0.466 | 0.345 |
|  | All | | 0.347 | 0.322 | 0.339 | 0.335 |

**Table S2.** Fairness evaluation results of different models in the skin disease identification task in terms of DI. DI: disparate impact; Mel: melanoma; MN: melanocytic nevi; BKL: benign keratosis-like lesions.

|  | | DI | | | |
| --- | --- | --- | --- | --- | --- |
|  |  | Mel. | MN | BKL | All |
| ChatGPT-4 | Sex | 0.985 | 0.838 | 0.862 | 0.934 |
|  | Age group | 0.966 | 0.973 | 0.867 | 0.98 |
| LLaVA-1.6 | Sex | 0.857 | 0.857 | 0.949 | 0.911 |
|  | Age group | 0.878 | 0.944 | 0.99 | 0.954 |
| Swin-B | Sex | 0.946 | 0.998 | 0.995 | 0.997 |
|  | Age group | 0.984 | 0.966 | 0.997 | 0.989 |
| VGG16 | Sex | 0.61 | 0.977 | 0.962 | 0.928 |
|  | Age group | 0.708 | 0.983 | N/A | 0.932 |
| ResNet50 | Sex | 0.938 | 0.977 | 0.691 | 0.96 |
|  | Age group | 0.862 | 0.88 | 0.6 | 0.941 |
| Model Derm | Sex | 0.719 | 0 | 0.943 | 0.69 |
|  | Age group | 0.89 | 0.956 | 0.941 | 0.884 |
| Random Model | Sex | 0.889 | 0.997 | 0.917 | 0.958 |
|  | Age group | 0.922 | 0.981 | 0.905 | 0.983 |

**Table S3.** Fairness evaluation results of different models in the skin disease identification task in terms of EOP. EOP: equal opportunity; Mel: melanoma; MN: melanocytic nevi; BKL: benign keratosis-like lesions.

|  | | EOP | | | |
| --- | --- | --- | --- | --- | --- |
|  |  | Mel. | MN | BKL | All |
| ChatGPT-4 | Sex | 0.011 | 0.084 | 0.033 | 0.033 |
|  | Age group | 0.038 | 0.018 | 0.053 | 0.028 |
| LLaVA-1.6 | Sex | 0.057 | 0.084 | 0.042 | 0.053 |
|  | Age group | 0.135 | 0.076 | 0.015 | 0.063 |
| Swin-B | Sex | 0.035 | 0.002 | 0.004 | 0.003 |
|  | Age group | 0.041 | 0.089 | 0.028 | 0.021 |
| VGG16 | Sex | 0.215 | 0.021 | 0.004 | 0.035 |
|  | Age group | 0.212 | 0.066 | 0.088 | 0.138 |
| ResNet50 | Sex | 0.014 | 0.019 | 0.094 | 0.017 |
|  | Age group | 0.049 | 0.157 | 0.163 | 0.102 |
| Model Derm | Sex | 0.081 | 0.31 | 0.037 | 0.129 |
|  | Age group | 0.1 | 0.025 | 0.269 | 0.122 |
| Random Model | Sex | 0.032 | 0.001 | 0.03 | 0.014 |
|  | Age group | 0.056 | 0.054 | 0.154 | 0.017 |

**Table S4.** Fairness evaluation results of different models in the skin disease identification task in terms of EOD. EOD: equalized odds; Mel: melanoma; MN: melanocytic nevi; BKL: benign keratosis-like lesions.

|  | | EOD | | | |
| --- | --- | --- | --- | --- | --- |
|  |  | Mel. | MN | BKL | All |
| ChatGPT-4 | Sex | 0.043 | 0.177 | 0.049 | 0.047 |
|  | Age group | 0.138 | 0.344 | 0.249 | 0.042 |
| LLaVA-1.6 | Sex | 0.087 | 0.19 | 0.06 | 0.08 |
|  | Age group | 0.193 | 0.39 | 0.09 | 0.089 |
| Swin-B | Sex | 0.058 | 0.019 | 0.008 | 0.004 |
|  | Age group | 0.116 | 0.294 | 0.113 | 0.031 |
| VGG16 | Sex | 0.216 | 0.042 | 0.009 | 0.05 |
|  | Age group | 0.333 | 0.323 | 0.315 | 0.207 |
| ResNet50 | Sex | 0.075 | 0.044 | 0.102 | 0.025 |
|  | Age group | 0.222 | 0.465 | 0.413 | 0.152 |
| Model Derm | Sex | 0.182 | 0.338 | 0.061 | 0.18 |
|  | Age group | 0.2 | 0.366 | 0.411 | 0.167 |
| Random Model | Sex | 0.084 | 0.085 | 0.031 | 0.021 |
|  | Age group | 0.17 | 0.483 | 0.359 | 0.025 |

## **S2. Supplementary Text**

### **S2.1. Supplementary Evaluation Metrics**

$\hat{Y}$, $Y$, $A$ and $n$ represent the predicted labels, true labels, sensitive attributes, and the number of sensitive attributes, respectively.

**Disparate Impact (DI)**

$$\begin{aligned} {DI}_{ij}=\min\left( \frac{P\left( \hat{Y}=1 | Y=1,A=i \right)}{P\left( \hat{Y}=1 | Y=1,A=j \right)},\frac{P\left( \hat{Y}=1 | Y=1,A=j \right)}{P\left( \hat{Y}=1 | Y=1,A=i \right)} \right), i, j=0,\ldots, n-1\#\left( S1 \right) \end{aligned}$$

$$\begin{aligned} DI=max{DI}_{ij}\#\left( S2 \right) \end{aligned}$$

**Equal opportunity (EOP)**

$$\begin{aligned} EOP=\frac{2}{n(n-1)}\sum_{i=0}^{n-2} \sum_{j=i+1}^{n-1} (\left| P\left\{ \hat{Y}=1 | Y=1,A=i \right\}-P\left\{ \hat{Y}=1 | Y=1,A=j \right\} \right|)\#\left( S3 \right) \end{aligned}$$

**Equalized odds (EOD)**

$$\begin{aligned} EOD=\frac{2}{n(n-1)}\sum_{i=0}^{n-2} \sum_{j=i+1}^{n-1} \sum_{y=0}^{1} (\left| P\left\{ \hat{Y}=1 | Y=y,A=i \right\}-P\left\{ \hat{Y}=1 | Y=y,A=j \right\} \right|)\#\left( S4 \right) \end{aligned}$$
